# A Systematic Evaluation of Ensemble Learning Methods for Fine-Grained Semantic Segmentation of Tuberculosis-Consistent Lesions in Chest Radiographs

**DOI:** 10.3390/bioengineering9090413

**Published:** 2022-08-24

**Authors:** Sivaramakrishnan Rajaraman, Feng Yang, Ghada Zamzmi, Zhiyun Xue, Sameer K. Antani

**Affiliations:** National Library of Medicine, National Institutes of Health, Bethesda, MD 20892, USA

**Keywords:** bone suppression, chest X-rays, deep learning, ensemble, stacking, segmentation, tuberculosis

## Abstract

Automated segmentation of tuberculosis (TB)-consistent lesions in chest X-rays (CXRs) using deep learning (DL) methods can help reduce radiologist effort, supplement clinical decision-making, and potentially result in improved patient treatment. The majority of works in the literature discuss training automatic segmentation models using coarse bounding box annotations. However, the granularity of the bounding box annotation could result in the inclusion of a considerable fraction of false positives and negatives at the pixel level that may adversely impact overall semantic segmentation performance. This study evaluates the benefits of using fine-grained annotations of TB-consistent lesions toward training the variants of U-Net models and constructing their ensembles for semantically segmenting TB-consistent lesions in both original and bone-suppressed frontal CXRs. The segmentation performance is evaluated using several ensemble methods such as bitwise- AND, bitwise-OR, bitwise-MAX, and stacking. Extensive empirical evaluations showcased that the stacking ensemble demonstrated superior segmentation performance (Dice score: 0.5743, 95% confidence interval: (0.4055, 0.7431)) compared to the individual constituent models and other ensemble methods. To the best of our knowledge, this is the first study to apply ensemble learning to improve fine-grained TB-consistent lesion segmentation performance.

## 1. Introduction

Tuberculosis (TB) continues to remain the primary cause of ill-health and mortality across the world according to the recent 2021 global reports on TB from the World Health Organization (WHO) [1]. Pulmonary infection from the *Mycobacterium* is reported to affect all age groups and sexes. Early screening and diagnosis would therefore prove to be critical to improving chances of survival and patient care. 

While CT imaging is the preferred diagnostic imaging technique because of its sensitivity in detecting TB, it has several limitations such as reduced access in low and medium-resourced regions, high cost, high radiation dose, lack of portability, and increased need for frequent sanitation, among others [2]. Therefore, CXR imaging continues to be the most widely used examination for pulmonary TB-related disease screening [3], particularly in developing countries with limited technical and human resources. However, factors including lack of human expertise in interpreting CXR images and reallocation of services from dealing with TB to responding to evolving pulmonary infections like COVID-19, among others, have necessitated developing automated methods for TB screening and diagnosis. Under these circumstances, artificial intelligence (AI)-based semantic segmentation methods using machine learning (ML)/deep learning (DL) algorithms [4,5] could help segment TB-consistent manifestations and thereby supplement clinical decision-making.

Semantic segmentation methods associate each image pixel with a class label. Automatic DL-based algorithms are shown to deliver superior performance in delineating and identifying disease-specific manifestations in CXRs, particularly TB [6,7]. They can help supplement human expertise for clinical decision-making thereby facilitating prompt referrals and subsequently improving patient care. However, the performance of DL models is observed to scale with the availability and characteristics of data and computational resources. Unlike natural images, it is rather difficult to obtain medical images and the associated disease-specific annotations due to various factors including varying image acquisition methods, varying disease pathogenicity across the world, inter- and intra-observer variability in annotating the disease-specific regions of interest (ROIs), the granularity of annotations, and the availability of data and annotations for research due to patient privacy and other ethical concerns.

### 1.1. Related Literature

A major limitation of current DL algorithms is that they use coarse bounding-box annotations of TB-consistent lesions for training and validating the models [8]. This might result in including a considerable fraction of false-positive and false-negative pixels in the annotations since the TB infection-specific ROIs are relatively small and there is variability in the granularity of expert annotations used to train the models. To the best of our knowledge, until the time of writing this manuscript, no CXR dataset accompanied by fine-grained annotations of TB-consistent lesions is publicly available to train and evaluate DL models.

DL models learn through stochastic backpropagation [9]. Due to the varying architecture and hyperparameters of the model and the stochastic nature of learning, these models may converge to different local optima. Ensemble learning is an established ML paradigm that seeks to improve robustness and accuracy by combining the predictions of several models [10]. Several methods of ensemble learning (e.g., averaging, bagging, boosting, and stacking) are shown to deliver superior performance in medical image segmentation tasks using CXRs, particularly for lung segmentation. The authors of [11] performed an averaging ensemble of the predictions of the U-Net [12] and DeepLabV3+ models to segment lungs in CXRs. Their proposed method achieved a segmentation accuracy of 98.6% using the Japanese Radiological Scientific Technology (JRST) [13] and Shenzhen CXR [14] datasets. In another study [15], the authors proposed an ensemble DeepLabV3+ based architecture to segment lungs in the Shenzhen CXR collection and achieved an Intersection-Over-Union (IoU) score of 0.97. Ensemble methods were applied to segment pneumothorax-consistent regions [16] in CXRs. The authors used various ImageNet-pretrained encoder backbones in the U-Net model and performed a weighted averaging ensemble of their predictions to segment pneumothorax-consistent regions with a dice score of 0.906. However, until the time of writing this manuscript, no literature exists in evaluating the gains achieved through ensemble learning, particularly applied to segmenting TB-consistent ROIs in CXRs.

### 1.2. Contributions of the Study

The contributions of this study are summarized as follows:

1. This study proposes to use fine-grained annotations of TB-consistent lesions to train and evaluate the performance of variants of U-Net-based segmentation models.
2. The gains achieved through constructing an ensemble of the trained models were evaluated to demonstrate further improvement in the robustness and performance of the segmentation algorithms.

**Figure 1:**
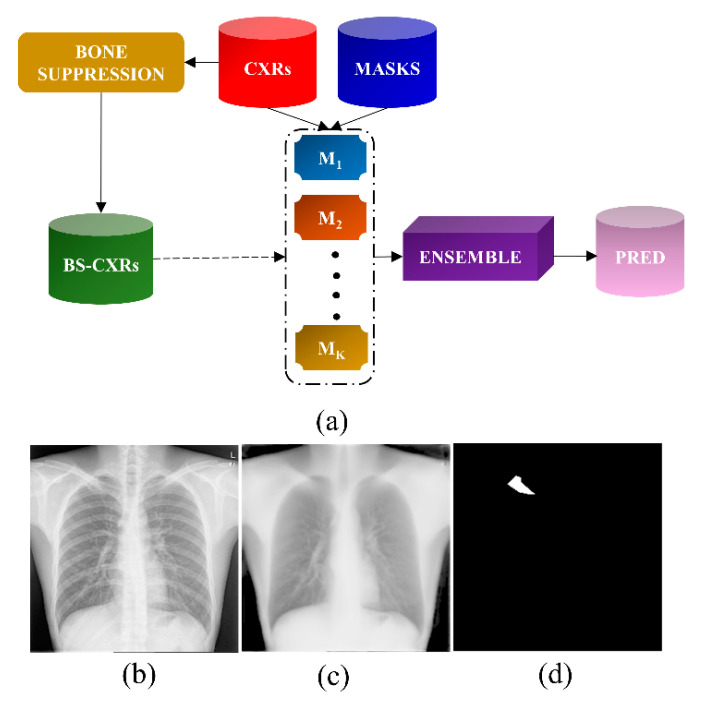
Block diagram of the proposal. (**a**) Process pipeline in training individual U-Net models and constructing ensembles to arrive at the final prediction; (**b**–**d**) show a sample CXR, its bone-suppressed counterpart, and the ground truth TB-consistent lesion mask, respectively.

A block diagram that summarizes our study is shown in Figure 1. In the first step, the feature pyramid network (FPN)-based model with the EfficientNet-B0 encoder backbone proposed in our previous study [17] was to suppress bones in the Shenzhen TB CXR collection. Then, U-Net models with varying ImageNet-pretrained encoder backbones, viz., ResNet-34 [18], Inception-V3 [19], DenseNet-121 [20], EfficientNet-B0 [21], and SE-ResNext-50 [22] were trained and evaluated on the original and bone-suppressed CXRs for segmenting TB-consistent lesions. The predictions of the top-K (K = 3, 4, 5) models were used to construct ensemble predictions using several bitwise operations, viz., bitwise-AND, bitwise-OR, and bitwise-MAX. A stacking ensemble was constructed by concatenating the features extracted from the penultimate layer of the top-K models and training a fully-convolutional meta-learner to optimally combine these features and improve segmentation performance.

The datasets used, their characteristics, model architecture, loss functions, and evaluation metrics are discussed in Section 2, related results and discussions are elaborated in Section 3, and the conclusion and scope for future research are discussed in Section 4.

## 2. Materials and Methods

### 2.1. Datasets

The Shenzhen TB CXR [14] dataset, which contains 662 de-identified CXRs including 336 TB cases and 326 normal cases was used in this study. The number of CXRs in the train, validation and test sets are shown in Table 1. TB cases were either microbiologically confirmed, or with clinical symptoms and imaging appearance consistent with TB, and positive response to anti-TB medication while excluding other causes. The CXRs were collected from patients at the Shenzhen No.3 hospital in Shenzhen, China. The use of these CXRs is exempted from IRB review (OHSRP#5357) by the National Institutes of Health (NIH) and is made publicly available. CXRs manifesting TB-consistent abnormalities were annotated by two radiologists from the Chinese University of Hong Kong using the Firefly annotation tool (https://cell.missouri.edu/software/firefly/, accessed on 3 December 2021). The labeling was initially conducted by a junior radiologist, then the labels were all checked by a senior radiologist, with a consensus reached for all cases. Of the 336 CXRs that carry TB labels, the radiological signs consistent with TB were observed only in 330 CXRs. The annotations were prepared in JavaScript object notation syntax (JSON) format. They were also prepared as separate grayscale mask images showing abnormal ROIs.

The CXRs and their associated masks were resized to 256 × 256 spatial dimensions to reduce computational complexity. The resized CXRs and masks were split into 70% for training (n = 231), 20% for validation (n = 66), and 10% for testing (n = 33). The CXRs used for training were further augmented offline using the Augmentor tool [23] using affine transformations including mirroring, rotation in the range [5,10], and zooming in the range [0.8, 1.4] to create 2000 additional CXRs and their associated masks. Therefore, there were 2231 CXR images in the training set after augmentation.

### 2.2. Model Architecture

#### 2.2.1. Bone Suppression

The bones in the Shenzhen TB CXR collection were suppressed using an FPN-based model [24] with an EfficientNet-B0 encoder backbone, which was used in our previous study [17]. The bottom-up pathway extracts image features at multiple scales. The spatial resolution decreases with increasing depth and the semantic value of the layers increases while detecting high-level structures. The top-down pathway constructs high-resolution layers from the semantically rich layers at each scale in the bottom-up pathway. The final layer of the models consists of a convolutional layer with Sigmoidal activation to predict bone-suppressed CXRs. The original CXRs and their bone-suppressed counterparts were trained on the augmented NIH-CC-DES-Set 2 dataset and tested with the CXR image pairs in the NIH-CC-DES-Set 1 dataset [17]. The learning rate was reduced whenever no improvement in the validation performance was observed for the subsequent five epochs. Callbacks were used to store model checkpoints and stopped training when the performance on the validation set began to degrade. The models were trained on an Ubuntu Linux system with NVIDIA GeForce GTX 1080 Ti graphics card using the Keras framework with Tensorflow backend and CUDA dependencies for accelerating the GPUs.

#### 2.2.2. Segmentation of TB-Consistent Lesions

The U-Net variants with ImageNet-pretrained encoder backbones, viz., ResNet-34, Inception-V3, DenseNet-121, EfficientNet-B0, and SE-ResNext-50 were trained to segment the TB-consistent lesions in the CXRs. U-Net has a U-shaped architecture with an encoder followed by a decoder network. The various ImageNet-pretrained models, aforementioned, used in the encoder/contracting path encode the input CXRs into feature representations at multiple scales. The number of feature channels gets doubled with each down-sampling step. The decoder/expanding path up-samples the feature maps to project the low-resolution features into the high-resolution pixel space. The skip connections/concatenations ensure that the low-level information was shared between the input and output, thereby adding information that might be lost because of the down-sampling on the encoder side of the network. The final convolutional layer in the decoder network with Sigmoidal activation predicts the masks. The U-Net models were trained on the augmented Shenzhen TB CXRs and their corresponding TB-consistent lesion masks (from Table 1) using an Adam optimizer with an initial learning rate of 1 × 10^−4^. Callbacks were used to store model checkpoints. The learning rate was reduced whenever the validation performance ceased to improve. The best-performing checkpoint with the validation data was used to predict the test data and generate masks.

#### 2.2.3. Ensemble Learning

**Figure 2:**
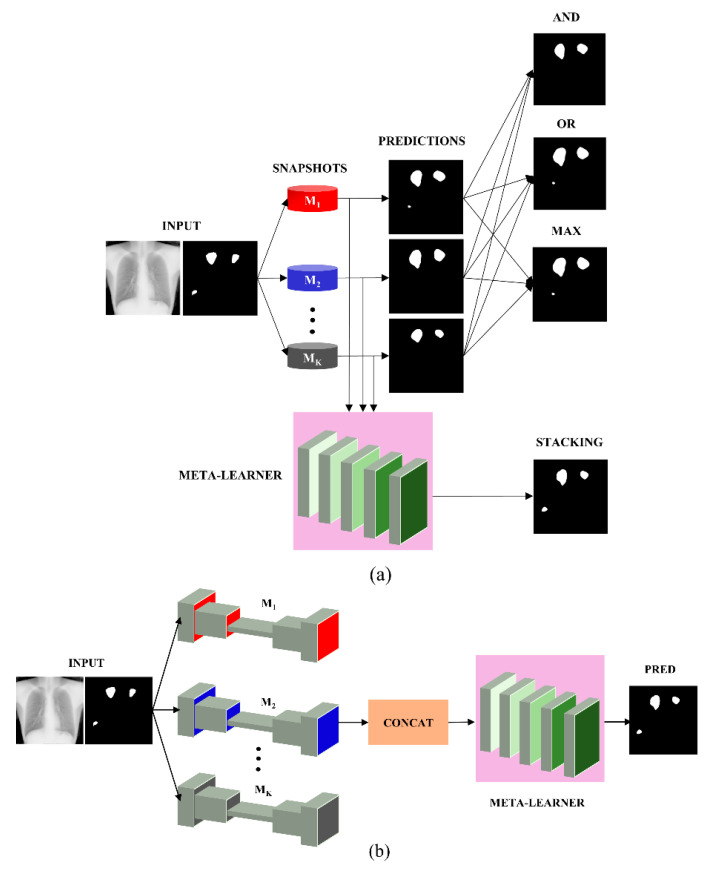
Ensemble strategies using the predictions of top-K (K = 3, 4, 5) models. (**a**) shows the method flowchart showing bitwise-OR, bitwise-AND, bitwise-MAX, and stacking ensemble outputs and (**b**) shows the method flowchart detailing the stacking process.

The predictions of the top-K (K = 3, 4, 5) models were combined using the following ensemble methods: (i) bitwise-AND, (ii) bitwise-OR, (iii) bitwise-MAX, and (iv) Stacking, as illustrated in Figure 2. For bitwise operations, a pixel-wise comparison of the predicted masks by the constituent models was performed to construct the final prediction. For a bitwise-AND ensemble, the pixel in the final prediction was turned on only if the corresponding pixels in the predictions from the top-K models were greater than 0. As for a bitwise-OR ensemble, the pixel in the final prediction was turned on if even one of the corresponding pixels in the predictions from the top-K models was greater than 0. The bitwise-MAX was computed across the masks predicted by the top-K models to turn on the corresponding pixel in the final ensemble prediction, otherwise, the pixels were set to 0.

A stacking ensemble, as illustrated in Figure 2b, was further constructed using the top-K (K = 3, 4, 5) models as follows: (i) Each of the top-K models was initialized with their trained weights. (ii) The features from the penultimate layer of the top-K models were extracted and concatenated. (iii) A fully-convolutional meta-learner performed second-level learning on these concatenated features. (iv) The trainable weights of the top-K models were frozen and only the fully-convolutional meta-leaner was trained on the concatenated features. (v) The fully convolutional, second-level meta-learner consisted of five convolutional layers. The number of filters in the 1st, 2nd, 3rd, 4th, and 5th convolutional layers were 256, 128, 64, 32, and 1, respectively. (vi) All convolutional filters except for those in the final convolutional layer were 3 × 3 dimensions and they used ReLU activation. (vii) The final convolutional layer with sigmoidal activation and one filter of dimension 1 × 1 predicted the masks. (viii) The predicted masks were compared to the ground-truth (GT) masks to evaluate segmentation performance.

The stacking ensemble was trained with an Adam optimizer with a learning rate of 1 × 10^−3^ to minimize the proposed loss function discussed in Equation (9). Callbacks were used to store model checkpoints whenever the validation loss decreased. The learning rate was reduced whenever the validation loss ceased to improve. The training was stopped when this loss plateaued. The stacking ensemble model weights that delivered the best validation performance were further used to predict the test set and the performance was recorded in terms of dice, Intersection over Union (*IoU*), and area under the precision (P)-recall (R) curve (AUPRC) metrics discussed in Section 2.2.4.

#### 2.2.4. Loss Functions and Evaluation Metrics

An Adam optimizer with an initial learning rate of 1 × 10^−3^ was used to reduce a mixed loss function given by Equation (1) to train the bone suppression model. Here, MS-SSIM denotes the multi-scale structural similarity index measure and MAE denotes the mean absolute error. The value of *α* = 0.84 and *β* = 0.16 was empirically determined to deliver superior performance.

(1) Mixed−loss=α×MS−SSIM+β×MAE

The U-Net-based segmentation models were evaluated in terms of *IoU*/Jaccard score, dice/F1 score, and area under the precision (*P*)-recall (*R*) curve. The *IoU* metric is widely used in evaluating semantic segmentation tasks. It is given by Equations (2) and (3).

(2) IoU=TP/(TP+FP+FN)

(3) $$IoU_{loss}=1-IoU$$

Here, *TP*, *FP*, and *FN* denote the true positives, false positives, and false negatives, respectively, in segmenting the TB-consistent lesions for a given *IoU* threshold. The dice score is another widely used segmentation evaluation metric, given by Equations (4) and (5). Higher values of the *IoU* and dice score denote improved similarity between the predicted and GT masks.

(4) Dice score=2×TP/(2×TP+FP+FN)

(5) $$Dice_{loss}=1-Dice\;score$$

The mean average precision (mAP) is measured as the area under an 11-point interpolated PR curve, given by Equations (6)–(8). Here, *Precision* (*P*) measures the accuracy of predictions, and *Recall* (*R*) measures how well the model identifies all the TPs. The *R* values were segmented evenly into 11 parts, i.e., {0, 0.1, 0.2, 0.3, …, 0.9, 1.0} and the *mAP* is calculated by measuring the AUPRC. The *IoU* threshold was fixed as 0.5.

(6) $$Precision\left(P\right)=\frac{TP}{TP+FP}$$

(7) $$Recall\;\left(R\right)=\frac{TP}{\left(TP+FN\right)}$$

(8) $$mAP=\frac{1}{11}\sum_{Recall_{i}}Precision\left(Recall_{i}\right)$$

For segmenting the TB-consistent lesions and to enforce all models to have a high recall, the boundary uncertainty (BU) evaluation [25] was included while minimizing the Focal Tversky (*FT*) loss function [26], given by Equation (9). The *FT* loss is parameterized by *γ* to balance between the majority background and minority TB-consistent lesion (ROI) pixels. The value TI, given by Equation (10), denotes the Tversky index (*TI*) function [26] which generalizes the dice score. Here, c denotes the minority TB-consistent ROI.

(9) $$FT_{loss}{\left(p,p^{\prime }\right)}_{c}=\sum_{c}\left(1-TI_{c}^{\gamma }\right)$$

(10) $$TI\left(p,p^{\prime }\right)=\frac{pp^{\prime }}{pp^{\prime }+\lambda \left(1-p\right)p^{\prime }+\left(1-\lambda \right)p\left(1-p^{\prime }\right)}$$

Here, *λ*∈ [0, 1]. When *λ* = 0.5, the equation simplifies to the regular dice score. Higher values of *λ* will penalize the FNs more than the FPs. That is, with higher values of *λ*, the FNs will be kept low with increasing recall since we were concerned with how well the model identifies all the TPs. The value of *λ* = 0.7 and γ = 0.75 was fixed after extensive empirical evaluations.

In a binary segmentation problem, each pixel *t* in the GT mask, at location *x*, is assigned a hard class label as shown in Equation (11).

(11) $$t_{x}:\{\begin{array}{c} t=1,ifx\in ₣\\ t=0,ifx\notin ₣ \end{array}$$

Here, ₣ denotes the target. While evaluating BU, the hard labels 0 and 1 are converted into soft labels to represent probabilistic scores.

(12) $$t_{x}:\{\begin{array}{c} t\leq 1,ifx\in ₣\\ t\geq 0,ifx\notin ₣ \end{array}$$

(13) $$t_{x\notin ₣}\leq t_{x\in ₣}$$

Equations (12) and (13) explain that the values closer to 1 and 0 denote higher confidence in classifying the pixels as belonging to the TB disease-consistent ROI or background respectively. The soft labels are restricted only to the ROI boundaries to approximate the uncertainty in segmentation using morphological operators such as dilation (⨹) and erosion (⨺) [25]. Let *X* denote the input image of dimension *a × b*. The BU function performs dilation and erosion operations on the ROI boundaries at all positions by querying with a structural element *Y* of 3 × 3 spatial dimensions as shown in Equations (14) and (15). Probabilities are then assigned for the pixels on the ROI boundaries as shown in Equation (16).

(14) $$\left(X\;⨹\;Y\right)\left(x,y\right)=max_{i\in S1j\in S2}\;\left(X\left(x-i,y-j\right)+Y\left(i,j\right)\right)$$

(15) $$\left(X\;⨺\;Y\right)\left(x,y\right)=min_{i\in S1j\in S2}\;\left(X\left(x+i,y+j\right)-Y\left(i,j\right)\right)$$

(16) $$t_{x\in ₣}:\{\begin{array}{c} t=\zeta ,\;if\;t\;\in (\left(X⨹Y{)}_{n}-X\right)\\ t=\Omega ,\;if\;t\;\in (X-\left(X\;⨺Y{)}_{n}\right)\; \end{array}$$

Here, *n* = 1 represents the iterations for which the morphological operators are applied. The hyperparameters ζ and *Ω* denote the values for the soft labels that are exterior and interior to the ROI boundaries, respectively. When ζ = 1 and *Ω* = 0, the soft labels would converge to the original hard labels. After empirical evaluations, the value of ζ = 0.9 and *Ω* = 0.1 was fixed. This BU component was incorporated with the *FT* loss function to train the U-Net variants toward segmenting TB-consistent lesions.

#### 2.2.5. Statistical Analysis

The statistical significance was reported for the dice score with the hold-out test data. The 95% confidence intervals (CIs) were reported as the binomial Clopper–Pearson interval for the dice scores. The guidelines in [27] were followed to measure the p-values from the CIs.

## 3. Results and Discussion

**Figure 3:**
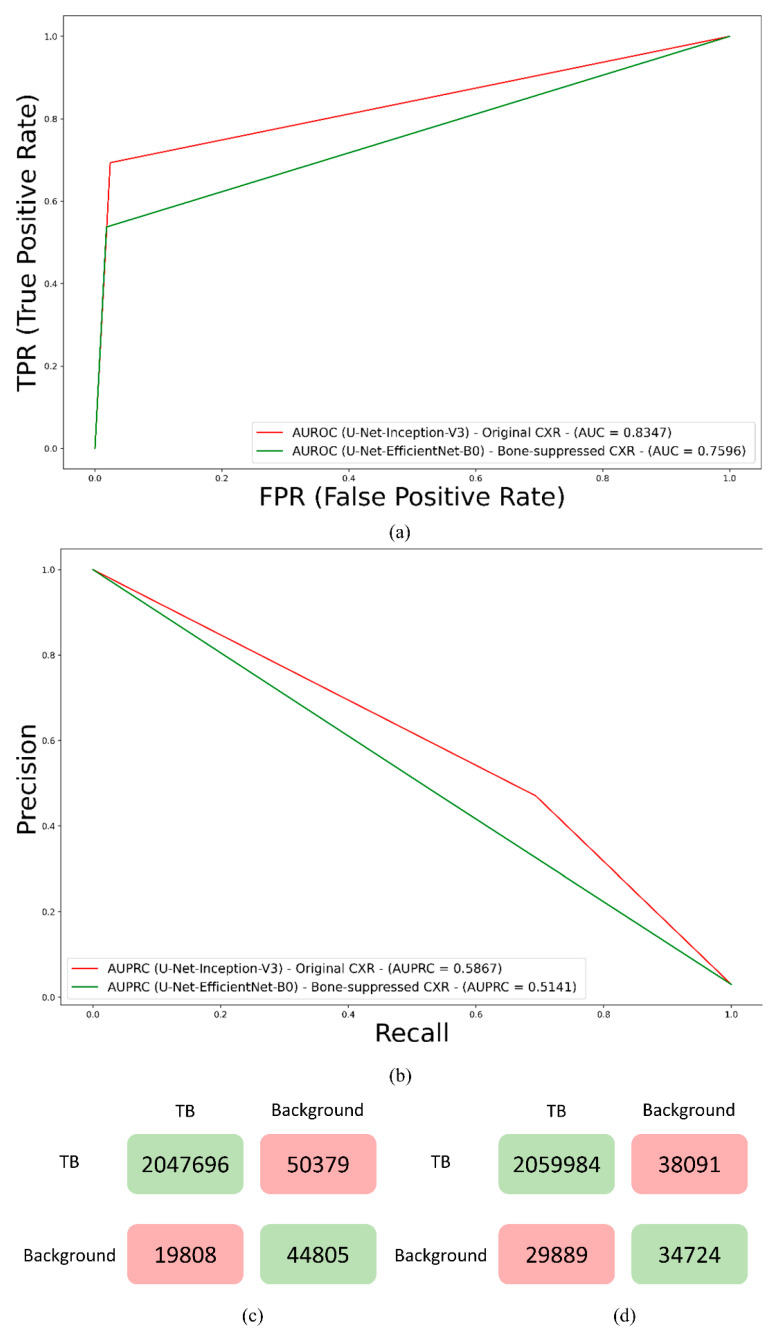
Performance of the top-performing U-Net with Inception-V3 encoder backbone trained on original CXRs and EfficientNet-B0 encoder backbone trained using bone-suppressed CXRs. (**a**) ROC curves; (**b**) PR curves; (**c**) Confusion matrix achieved with original CXRs, and (**d**) Confusion matrix achieved with bone-suppressed CXRs.

Recall that the U-Net models were trained on the original and bone-suppressed CXRs. Table 2 shows their TB-consistent ROI segmentation performance. Figure 3 shows the receiver-operating-characteristic (ROC) curves, PR curves, and confusion matrices achieved by the top-performing models trained on original and bone-suppressed CXRs, respectively. It was observed from Table 2 and Figure 3 that the U-Net models trained on the original CXRs outperformed those trained on bone-suppressed CXRs in terms of *IoU*, dice score, AUPRC, and area under the receiver-operating characteristic (AUROC) curve.

**Figure 4:**
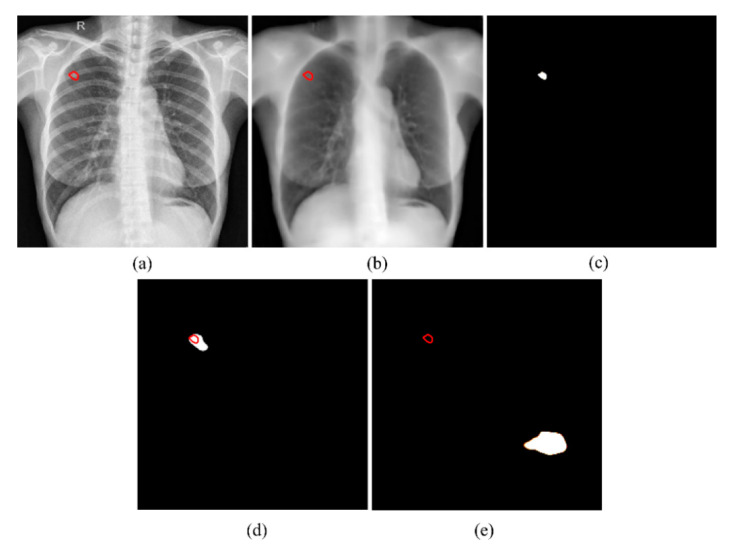
TB-consistent lesion segmentation performance using the U-Net with Inception-V3 encoder backbone trained on original CXRs and EfficientNet-B0 encoder backbone trained using bone-suppressed CXRs. (**a**) Sample original CXR image with TB-consistent lesion annotated in red; (**b**) Corresponding bone-suppressed CXR image with TB-consistent lesion annotated in red; (**c**) GT TB-consistent lesion mask; (**d**) Predicted mask using the Inception-V3 encoder backbone trained on original CXRs with overlapping GT annotations in red, and (**e**) Predicted mask using the EfficientNet-B0 encoder backbone trained using bone-suppressed CXRs with overlapping GT annotations in red.

This difference in performance was surprising and counter-intuitive since bone suppression would improve soft-tissue visibility in the image. However, we believe that it can be attributed to the following: (i) The FPN model with the EfficientNet-B0-based encoder was trained and evaluated on a different, sparse, bone-suppressed CXR collection from [17]. The model was trained in-house and not widely tested with cross-institutional datasets. This could have impacted the model’s generalization to other datasets due to the heterogeneities in X-ray acquisition and imaging protocols, variability in the overlying cardiopulmonary structures, and bone individualities such as previous fractures and other support devices in the CXR collections. The lack of generalization might have resulted in uneven suppression, brightness, and contrast changes, and irrelevant suppression of the soft tissues, which adversely impacted the detection of apical, central, and basal lesions, and subsequent disease-specific segmentation performance as with TB. With the increased availability of dual-energy subtraction (DES) CXRs, bone-suppressed images from the device could be used in the training process which could introduce sufficient data diversity, and therefore help train deeper model architectures to generalize to real-world data. Studies in the literature report that the soft tissue projections obtained with DES systems are superior in quality compared to those generated by DL-based bone suppression methods [28]. Automated bone suppression methods are also shown to be lacking in preserving the frequency details in the original images, therefore small lesions may fade out and go unnoticed upon removing the overlying bony structures in the CXRs [28]. Such phenomena can be noticed in Figure 4. Here, unlike the top-performing U-Net with Inception-V3 encoder backbone trained on original CXRs, the top-performing U-Net model with EfficientNet-B0 encoder backbone trained using bone-suppressed CXRs failed to segment the TB-consistent lesion. These factors might have contributed to the reduction in segmentation performance using the bone-suppressed CXRs. Therefore, we used the models trained on original CXRs to construct ensemble predictions to further improve segmentation performance.

**Figure 5:**
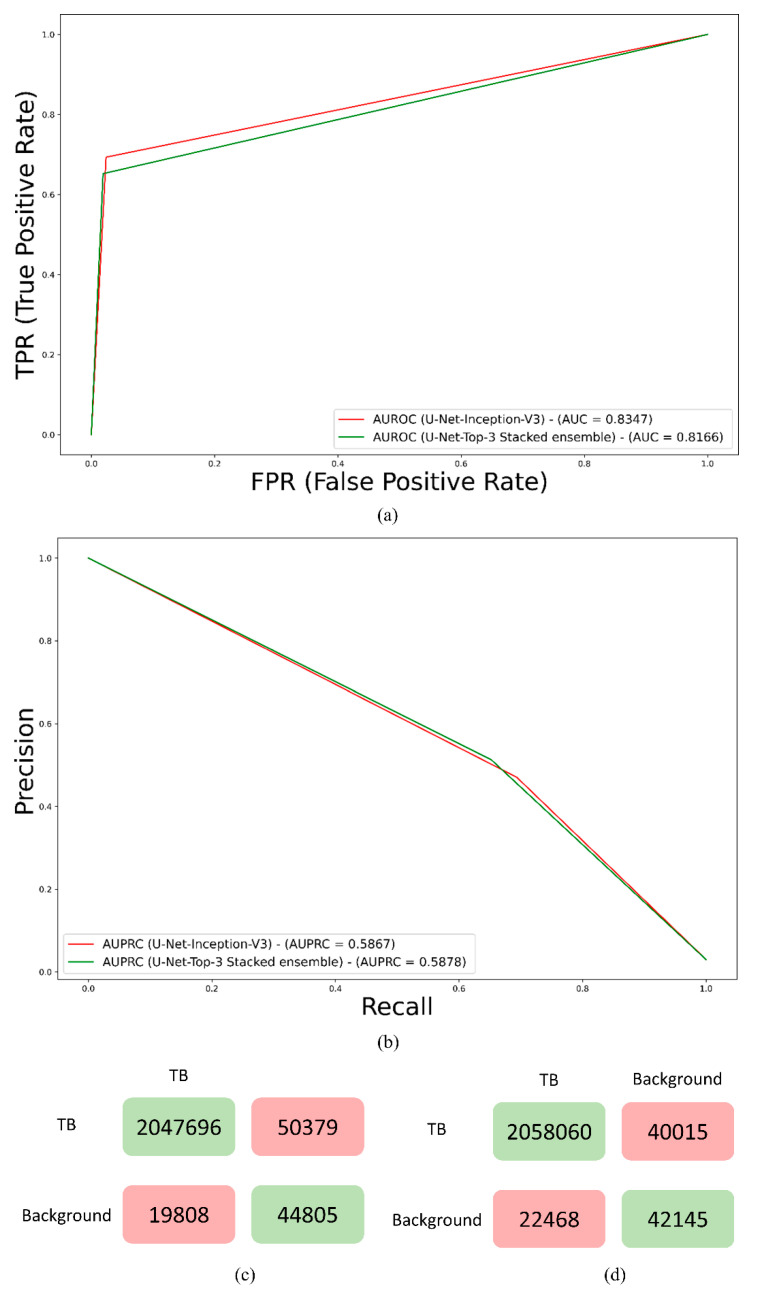
Performance of the U-Net with Inception-V3 encoder backbone and the stacking ensemble constructed using the top three performing models trained on original CXRs. (**a**) ROC curves; (**b**) PR curves; (**c**) Confusion matrix achieved with Inception-V3 encoder backbone, and (**d**) Confusion matrix achieved with the stacking ensemble.

**Figure 6:**
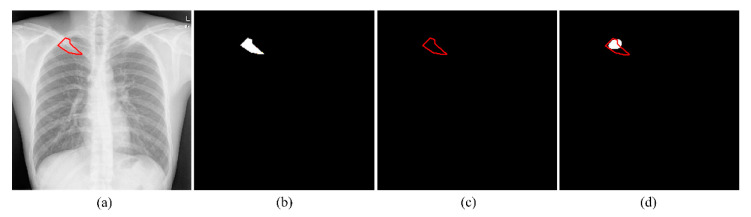
TB-consistent lesion segmentation performance using the U-Net with Inception-V3 encoder backbone and the stacking ensemble of the top-3 performing models. (**a**) Sample original CXR image with TB-consistent lesion annotated in red; (**b**) GT TB-consistent lesion mask; (**c**) Predicted mask using the Inception-V3 encoder backbone with overlapping GT annotations in red, and (**d**) Predicted mask using the stacking ensemble with overlapping GT annotations in red.

The TB-consistent lesion segmentation performance achieved by various ensemble methods was compared to the best-performing U-Net model with the Inception-V3 encoder backbone (baseline) as shown in Table 3. Figure 5 shows the ROC curves, PR curves, and confusion matrices obtained using the U-Net with Inception-V3 encoder backbone and the stacking ensemble constructed using the top-3 performing models trained on original CXRs. The predicted masks using these models for a sample CXR are shown in Figure 6.

The ensemble predictions were generated using the top-K (K = 3, 4, 5) models. It was observed that the stacking ensemble using the top three performing models, viz. the U-Net model with Inception-V3, ResNet-34, and EfficientNet-B0 encoder backbones, respectively, demonstrated superior segmentation performance in terms of IoU, dice score, and AUPRC, compared to the best-performing U-Net model with the Inception-V3 encoder backbone and other ensemble methods. It significantly outperformed (*p* < 0.05) the bitwise-OR and bitwise-MAX ensembles constructed using the top-K (K = 3, 4, 5) models in terms of the dice score. The TPs obtained with the stacking ensemble, i.e., the number of TB-consistent lesion pixels segmented correctly was higher than that achieved using the individual top-performing U-Net model with the Inception-V3 encoder backbone model. The improvement in performance using a stacking ensemble could be attributed to the fact that it used a second-level meta-learner that learned to optimally combine the features learned by the heterogeneous base learners, having different architectures, and learned diversified regions in the feature space to converge to their local optima, to output the final prediction.

## 4. Conclusions and Future Work

This study utilized fine-grained TB-consistent lesion annotations and demonstrated the efficacy of various ensemble methods for segmenting TB-consistent lesions in both original and bone-suppressed CXRs. However, the current proposal suffers from the following limitations: (i) CXRs with TB-consistent lesions that were used to train and evaluate the segmentation models were limited. Additional diversity in the training process could be introduced by using CXR data and annotations from multiple institutions. (ii) We used affine transformations to augment the data used for model training. Selecting an appropriate data augmentation technique is a challenging task and it depends on the characteristics of the data under study. Further, data augmentation could introduce more bias into model training if the original dataset contains the same. Therefore, it is critical to empirically identify an appropriate data augmentation strategy such that it creates data variability that can improve the ability of the models to optimally fit the training data while also generalizing it to real-world data. Future research could explore other advanced data augmentation methods including adversarial training, neural style transfer, and reinforcement learning, among others. (iii) We used the widely adopted U-Net architecture to segment TB-consistent lesions. Other advanced architectures including FPN, Link-Net, PSP-Net, Bi-SegNet [29], and Trilateral Attention Net [30], among others, and their ensembles could be trained for potential improvements in segmentation performance. With the advent of high-performance computing and storage solutions, ensemble models could be trained and deployed in the cloud to be used for real-time applications. The methods discussed in this study could be extended to a variety of natural and medical image recognition tasks [31,32,33,34].

## Figures and Tables

**Table 1:** Dataset and its respective patient-level train/validation/test splits.

Dataset	Train	Validation	Test
Shenzhen TB CXR	2231	66	33

**Table 2:** TB-consistent lesion segmentation performance using original (O) and bone-suppressed (BS) CXRs. Values in parentheses denote the 95% CIs for the dice score. Bold numerical values denote superior performance.

Models	IOU	Dice
ResNet-34 (O)	0.3599	0.5293 (0.3589, 0.6997)
ResNet-34 (BS)	0.3280	0.4640 (0.2938, 0.6342)
Inception-V3 (O)	**0.3896**	**0.5608 (0.3914, 0.7302)**
Inception-V3 (BS)	0.2525	0.4032 (0.2358, 0.5706)
DenseNet-121 (O)	0.2996	0.4611 (0.2910, 0.6312)
DenseNet-121 (BS)	0.2892	0.4486 (0.2789, 0.6183)
EfficientNet-B0 (O)	0.3453	0.5134 (0.3428, 0.6840)
EfficientNet-B0 (BS)	0.3381	0.5053 (0.3347, 0.6759)
SE-ResNext-50 (O)	0.3201	0.4850 (0.3144, 0.6556)
SE-ResNext-50 (BS)	0.2962	0.4570 (0.2870, 0.6270)

**Table 3:** TB-consistent lesion segmentation performance using various ensemble methods. The ensemble performance was compared to the top-performing U-Net model with the Inception-V3 encoder backbone (baseline). Values in parentheses denote the 95% CIs for the dice score. Bold numerical values denote superior performance.

Models	IOU	Dice
Inception-V3 (O)	0.3896	0.5608 (0.3914, 0.7302)
Top-3 ensemble		
Stacking	**0.4028**	**0.5743 (0.4055, 0.7431)**
Bitwise-AND	0.3829	0.5538 (0.3841, 0.7235)
Bitwise-OR	0.3558	0.5249 (0.3545, 0.6953)
Bitwise-MAX	0.3343	0.5011 (0.3305, 0.6717)
Top-4 ensemble		
Stacking	0.3962	0.5675 (0.3984, 0.7366)
Bitwise-AND	0.3534	0.5222 (0.3517, 0.6927)
Bitwise-OR	0.3088	0.4718 (0.3014, 0.6422)
Bitwise-MAX	0.2971	0.4581 (0.2881, 0.6281)
Top-5 ensemble		
Stacking	0.3974	0.5687 (0.3997, 0.7377)
Bitwise-AND	0.3534	0.5222 (0.3517, 0.6927)
Bitwise-OR	0.3088	0.4718 (0.3014, 0.6422)
Bitwise-MAX	0.2744	0.4306 (0.2616, 0.5996)

## Data Availability

The data supporting the reported results can be found at https://data.lhncbc.nlm.nih.gov/public/Tuberculosis-Chest-X-ray-Datasets/Shenzhen-Hospital-CXR-Set/Annotations/index.html, accessed on 20 May 2022.
